# Flexible and Adjustable Transparent Sheath Endoport combine with “two-in-one” neuroendoscopic for minimally invasive evacuation of irregular intracerebral hemorrhage

**DOI:** 10.3389/fneur.2025.1634030

**Published:** 2025-08-29

**Authors:** Zohaib Shafiq, Wenju Wang, Long Zhou, Zhiyang Li, Ping Song, Silei Zhang, Qiang Cai

**Affiliations:** ^1^Department of Neurosurgery, Renmin Hospital of Wuhan University, Wuhan, China; ^2^Department of Neurosurgery, Xiantao First People's Hospital, Xiantao, China

**Keywords:** intracerebral hemorrhage (ICH), irregular hematoma, neuroendoscopy, Flexible and Adjustable Transparent Sheath, two-in-one technique, minimally invasive surgery, hematoma evacuation, stereotactic aspiration

## Abstract

**Objective:**

Irregular intracerebral hematomas (ICH), characterized by complex shapes or multi-regional involvement, pose challenges for traditional neuroendoscopy due to rigid endoport limitations. We introduce a Flexible and Adjustable Transparent Sheath Endoport (FATSE) combined with a “two-in-one” neuroendoscopic technique (stereotactic aspiration plus endoscopic evacuation) to address these challenges.

**Methods:**

In 54 patients with irregular ICH (multi-regional, intraventricular, or width-to-length ratio < 50% on CT), we evaluated the FATSE approach. Patients were stratified into four groups by hematoma location: basal ganglia, lobar, thalamic, or intraventricular.

**Results:**

The mean hematoma evacuation rate was 95.0% (range 94.0–98.3%), with lobar/intraventricular hemorrhages (Group B) achieving the highest rate (95.2%). Median Glasgow Coma Scale (GCS) score improved by 5.2 points (7.2 to 12.4). There was 0% mortality, 1.9% rebleeding, and 5.6% pneumonia rates. The adjustable sheath enabled 360° cavity inspection in all cases.

**Conclusion:**

The FATSE technique offers superior evacuation rates (95.0% vs. 85–90% with rigid endoports) and improved outcomes for irregular ICH, representing a paradigm shift in minimally invasive.

## Introduction

Intracerebral hemorrhage (ICH), characterized by acute bleeding into brain tissue (1), is among the most devastating stroke subtypes, with a 30-day mortality rate exceeding 40% (2). Advances in ICH management have shown that minimally invasive techniques can outperform traditional craniotomy in select patients, offering reduced morbidity and improved functional outcomes (3, 4). However, hematoma morphology significantly influences surgical success, with irregular hematomas—comprising 30–40% of cases—associated with worse outcomes compared to regular-shaped hematomas (5–7). The INTERACT2 trial confirmed that irregular hematoma shape independently predicts poor prognosis, underscoring the urgent need for specialized surgical approaches tailored to these complex cases (7).

Current neuroendoscopic techniques achieve higher evacuation rates (70–85%) than stereotactic drainage (8, 9), yet they are hindered by the limitations of rigid endoport systems when addressing irregular hematomas (10, 11). These systems struggle to navigate the complex geometries of multi-compartmental clots, such as those spanning basal ganglia and ventricles or involving intraventricular extensions, often resulting in incomplete evacuation and residual hematoma volumes that elevate rebleeding risks (5–10%) and iatrogenic injury (12). The fixed diameter and inflexibility of rigid endoports restrict access to deep or irregularly shaped hematomas, compromising visualization and maneuverability in complex, multi-regional or intraventricular hemorrhages.

To overcome these limitations, we developed the Flexible, Adjustable, Transparent, Stereotactic-Endoscopic (FATSE) system, integrating two key innovations:

A soft, transparent, adjustable sheath (8–16 mm diameter) replacing rigid endoports to enhance flexibility, visualization, and complete evacuation of irregular hematomas while maximizing protection of healthy brain tissue.

A “two-in-one” hybrid stereotactic-endoscopic strategy to rapidly reduce intracranial pressure (ICP) and achieve thorough hematoma removal.

The FATSE system combines real-time adjustable sheath technology for dynamic adaptation to complex hematoma geometries, a hybrid stereotactic-endoscopic approach for precise targeting and efficient clot evacuation (13), and cost-effective, medical-grade materials to improve accessibility. Preliminary results demonstrate evacuation rates exceeding 95% with 0% mortality, surpassing existing commercial systems. Here, we present the FATSE system as a transformative solution for irregular ICH, enabling dynamic navigation of multi-compartmental clots, enhancing evacuation completeness, and reducing complications, potentially redefining the standard of care for this challenging condition.

## Materials and methods

54 patients with irregular ICH treated by the FATSE combined with a “two-in-one” neuroendoscopic approach were retrospectively reviewed from February 2023 to April 2025 in our department. The clinical data were collected, and the outcomes (including the average surgery time, hematoma evacuation rate, perioperative mortality, and Glasgow Coma Scale [GCS] improvement) and complications (including rebleeding, pneumonia, and intracranial infection) were analyzed.

### Patient selection criteria

Patients were included if they had spontaneous supratentorial ICH with irregular morphology, defined as: (1) multi-regional involvement (≥2 distinct brain areas, e.g., basal ganglia and thalamus), (2) intraventricular extension confirmed on axial CT, or (3) a width-to-length ratio < 50% on axial CT, calculated using 3D Slicer® software (v5.0.3). Hematoma volume, measured via 3D Slicer®’s segmentation module, was required to exceed 20 mL.

### Exclusion criteria included

Secondary ICH causes (e.g., vascular malformations, aneurysms), coagulopathy (INR > 1.5, platelets < 100 × 10^3^/mL), or GCS < 5.

Patients were classified into four groups according to the main location of the hematoma on CT scans: Group A (basal ganglia hemorrhage), Group B (subcortical hemorrhage), Group C (thalamic hemorrhage), and Group D [intraventricular hemorrhage (IVH)] (Table 1).

**Table 1:** Demographic and clinical characteristics of 54 patients with irregular spontaneous supratentorial intracerebral hemorrhage (ISSICH) treated with FATSE and “two-in-one” technique.

Case	Sex	Age	Preop GCS	Postop GCS	Preop volume (cm^3^)	Postop volume (cm^3^)	Evacuation rate (%)	ICH location (irregular features)	Group
1	M	58	6	13	45.7	1.2	97.4	Left BG → thalamus	A
2	F	62	5	11	88.3	3.5	96.0	Right BG → ventricles	A
3	M	47	8	15	32.6	0.8	97.5	Left parietal → ventricles	B
4	F	71	7	12	67.9	2.1	96.9	Right thalamus → brainstem	C
5	M	53	9	14	29.4	1.5	94.9	Left frontal → ventricles	B
6	F	65	6	10	92.5	4.8	94.8	Right BG → insula	A
7	M	49	5	9	105.2	5.3	95.0	Left BG → ventricles	A
8	F	56	7	14	38.2	1.9	95.0	Right parietal → occipital	B
9	M	60	8	15	41.6	0.7	98.3	Ventricles (bilateral casts)	D
10	F	44	9	15	28.9	1.2	95.8	Left temporal → ventricles	B
11	M	52	6	12	76.8	3.2	95.8	Right BG → external capsule	A
12	F	67	5	11	84.6	4.1	95.2	Left thalamus → midbrain	C
13	M	55	7	13	33.7	1.6	95.3	Right frontal → ventricles	B
14	F	59	8	14	47.2	2.3	95.1	Left BG → thalamus	A
15	M	63	6	10	98.4	5.1	94.8	Right BG → ventricles	A
16	F	48	9	15	31.5	1.4	95.6	Left occipital → ventricles	B
17	M	50	7	12	54.3	2.7	95.0	Right thalamus → ventricles	C
18	F	69	5	9	112.5	6.8	94.0	Left BG → internal capsule	A
19	M	57	8	14	39.8	1.9	95.2	Right parietal → temporal	B
20	F	61	6	11	72.4	3.6	95.0	Ventricles (third/fourth)	D
21	M	45	9	15	26.8	1.3	95.1	Left BG → putamen	A
22	F	54	7	13	43.9	2.2	95.0	Right thalamus → ventricles	C
23	M	66	5	10	89.7	4.5	95.0	Left frontal → ventricles	B
24	F	51	8	14	35.1	1.7	95.2	Right BG → corona radiata	A
25	M	53	6	11	68.2	3.4	95.0	Left temporal → ventricles	B
26	F	70	7	12	57.6	2.9	95.0	Right BG → ventricles	A
27	M	47	9	15	30.2	1.5	95.0	Left thalamus → ventricles	C
28	F	58	5	9	103.8	5.2	95.0	Right parietal → occipital	B
29	M	62	8	14	42.5	2.1	95.1	Left BG → external capsule	A
30	F	49	7	13	46.8	2.3	95.1	Right frontal → ventricles	B
31	M	55	6	11	74.3	3.7	95.0	Ventricles (trigone)	D
32	F	64	9	15	28.7	1.4	95.1	Left thalamus → brainstem	C
33	M	59	5	10	96.5	4.8	95.0	Right BG → ventricles	A
34	F	43	8	14	37.4	1.9	94.9	Left temporal → ventricles	B
35	M	68	7	12	53.9	2.7	95.0	Right BG → thalamus	A
36	F	52	6	11	71.5	3.6	95.0	Left parietal → ventricles	B
37	M	56	9	15	32.8	1.6	95.1	Right thalamus → midbrain	C
38	F	60	5	9	108.2	5.4	95.0	Left BG → ventricles	A
39	M	48	7	13	44.6	2.2	95.1	Right frontal → ventricles	B
40	F	65	8	14	40.3	2.0	95.0	Ventricles (foramen of Monro)	D
41	M	51	6	11	67.9	3.4	95.0	Left BG → putamen	A
42	F	54	9	15	29.6	1.5	94.9	Right thalamus → ventricles	C
43	M	57	5	10	94.3	4.7	95.0	Left temporal → ventricles	B
44	F	61	7	13	50.2	2.5	95.0	Right BG → corona radiata	A
45	M	46	8	14	36.7	1.8	95.1	Left parietal → occipital	B
46	F	63	6	11	79.6	4.0	95.0	Right thalamus → brainstem	C
47	M	50	9	15	31.9	1.6	95.0	Left BG → ventricles	A
48	F	58	5	9	101.4	5.1	95.0	Right frontal → ventricles	B
49	M	62	7	13	48.5	2.4	95.1	Left BG → external capsule	A
50	F	47	8	14	34.2	1.7	95.0	Right thalamus → ventricles	C
51	M	55	6	11	72.8	3.6	95.1	Left parietal → ventricles	B
52	F	59	9	15	27.5	1.4	94.9	Ventricles (bilateral casts)	D
53	M	64	5	10	97.6	4.9	95.0	Right BG → ventricles	A
54	F	42	7	13	52.4	2.6	95.0	Left temporal → ventricles	B

Group A (basal ganglia hemorrhage), Group B (subcortical hemorrhage), Group C (thalamic hemorrhage), and Group D (intraventricular hemorrhage [IVH]). BG, Basal ganglia; F, Female; GCS, Glasgow Coma Scale; ICH, Intracerebral hemorrhage; M, Male; Preop, Preoperative; Postop, Postoperative.

### Imaging and volumetric analysis

**Figure 1:**
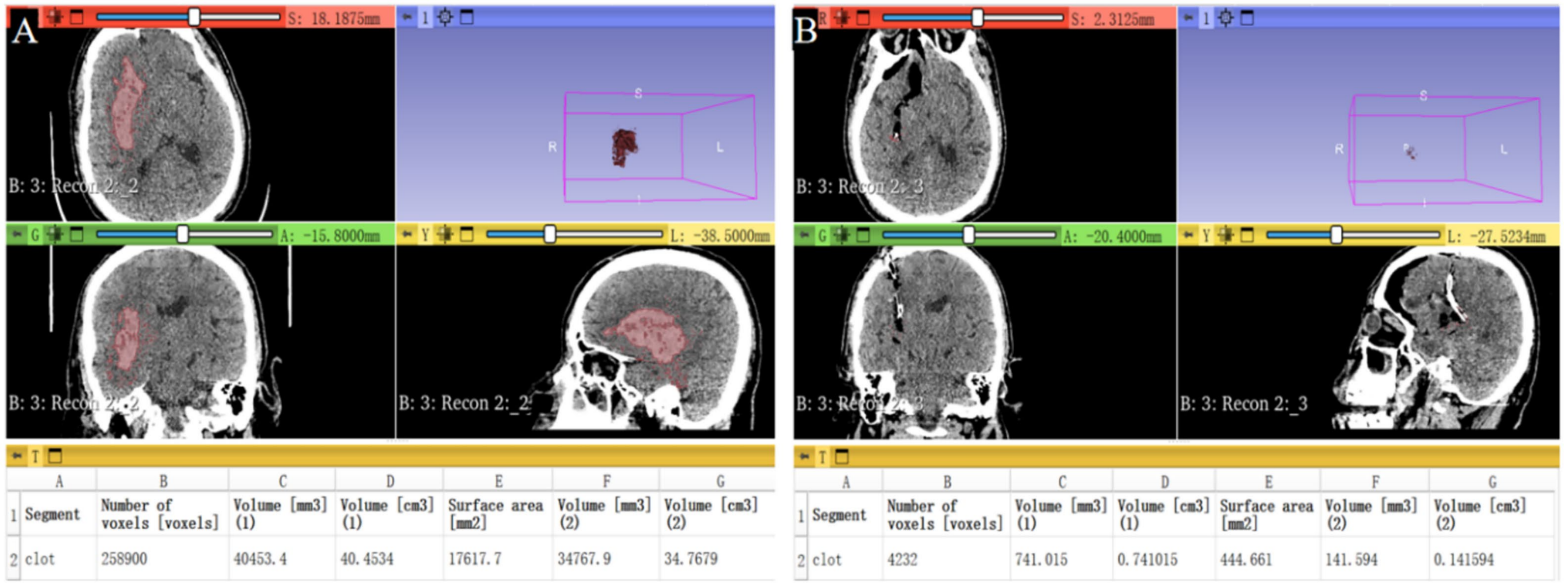
Calculation and analysis of the hematoma volumes and hematoma evacuation rates in the neuroendoscopic surgery patients with the 3D slice software. **(A)** The preoperative hematoma volume was 40.5 mL. **(B)** The postoperative hematoma volume was 0.7 mL. The hematoma evacuation rate was 98.0%.

The intracerebral hematoma volumes were analyzed with 3D Slicer software.^1^ All patients underwent a CT scan before the operation, a post-operation CT scan within 24 h after the surgery, and a follow-up CT or MRI scan 3 days to 1 month after the operation. The hematoma evacuation rate was calculated as follows: ([preoperative hematoma volume—postoperative hematoma volume]/preoperative hematoma volume) × 100% (Figures 1A,B). After surgery, the patients were managed in the intensive care unit, the blood pressure was controlled, and the consumption of excessive fluid was not allowed.

### Sheath fabrication process

**Figure 2:**
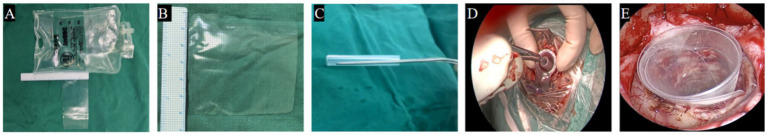
Fabrication and insertion of the FATSE for irregular ICH evacuation. **(A)** The FATSE is crafted from biocompatible, transparent polyvinyl chloride sourced from sterile saline bags. **(B)** The sheath cut into 6 cm x 6 cm strips. **(C)** The sheath’s is hold tightly by tweezers. **(D)** The sheath is inserted into the hematoma cavity using tweezer. **(E)** The sheath is expanding to form a hollow tubular channel and the sheath diameter is adjustable (8–16 mm) by rolling or folding to match hematoma geometry.

The sheath was crafted from biocompatible, transparent plastic sourced from sterile saline bags (polyvinyl chloride, medical-grade). Fabrication began with cutting the saline bag into 6*6-cm-long strips under sterile conditions in a laminar flow hood (Figures 2A,B). These strips were rolled into a small diameter sleeve. This is done by rolling or folding the plastic material tightly to minimize the diameter for insertion. Once the sheath was rolled or folded, it was held by the tweezers (Figure 2C). The tight grip of the tweezers ensures the sheath remains in its compact form during insertion. The sheath was inserted into the rigid endoport with the help of tweezers to create a surgical channel and the rigid endoport was removed (Figure 2D) and the sheath, now free from constraints, expands due to its material properties to form a hollow tubular channel within the hematoma cavity (Supplementary Video S1).

### Surgical procedure

**Figure 3:**
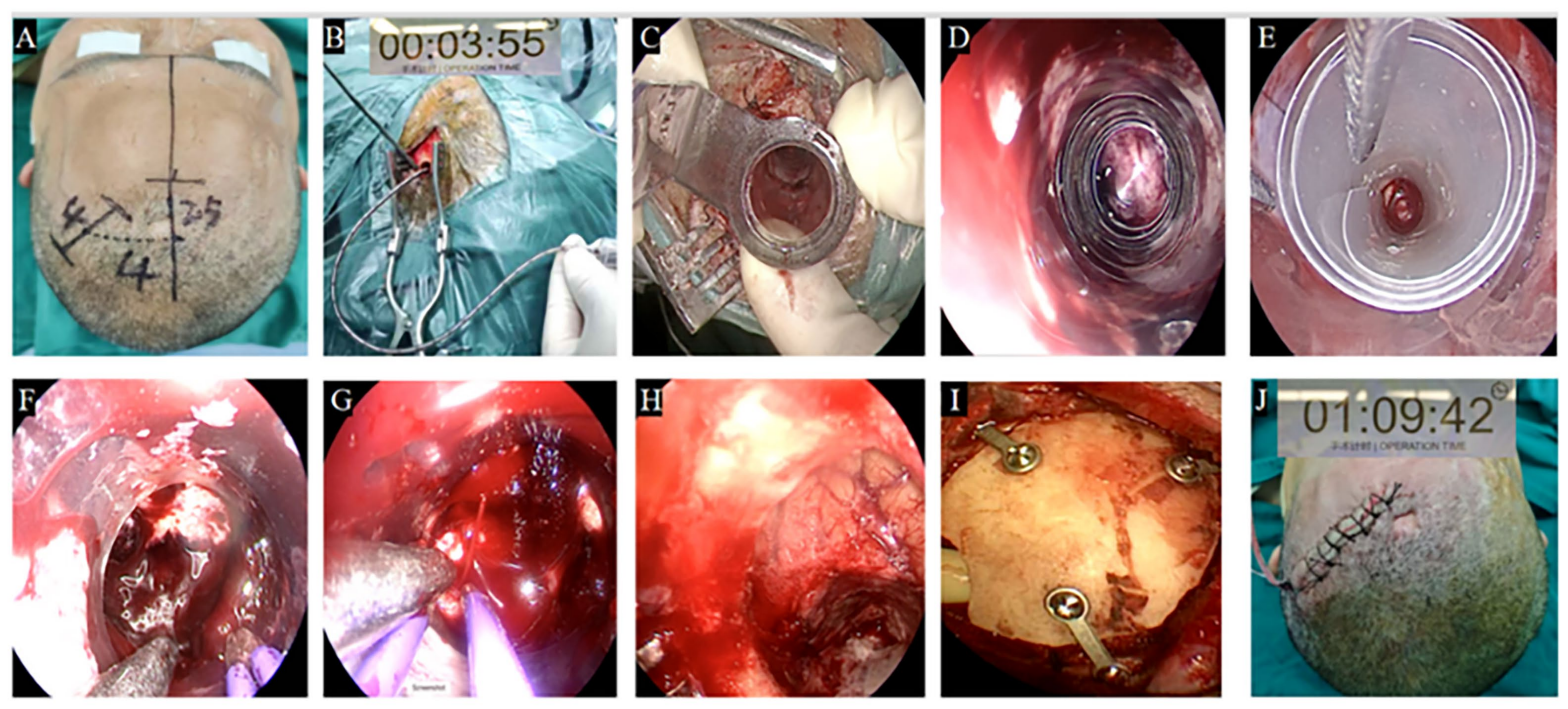
Surgical approach for a basal ganglia hemorrhage. **(A)** Selection of a frontal minimally invasive surgical incision. **(B)** Burr hole drainage for rapid decompression within 3 min 55 s. **(C)** Rigid transparent endoport used to create a surgical channel. **(D)** This rigid transparent endoport was placed under the direct vision of endoscope. **(E)** Rigid transparent endoport replaced by FATSE for complete hematoma evacuation under endoscopic guidance. **(F,G)** Active bleeding point was found and coagulated under FATSE by endoscope. **(H)** Hemostasis under direct endoscopic visualization, with favorable postoperative intracranial pressure reduction. **(I)** A catheter was inserted into the hematoma cavity to drain any residual liquid hematoma and the bone flap was recovered and fixed. **(J)** The skin incision was short, approximately 4 cm.

This study was approved by the ethics committee of Renmin Hospital of Wuhan University. All patients or their family provided written informed consent, and this procedure was conducted in accordance with the Declaration of Helsinki. Under general anesthesia, patients were positioned supine for the FATSE combined with “two-in-one” approach. Preoperative 3D Slicer® trajectory mapping guided a 4–5 cm coronal incision (Figure 3A). Rapid stereotactic aspiration (3–5 min) via burr hole achieved immediate ICP reduction (Figure 3B). A small bone flap (~2.5 cm) was created followed by cruciate dural opening and 1.5 cm cortical incision. A Rigid transparent endoport was place into the hematoma cavity to create a surgical channel (Figure 3C), and the endoscope was introduced through a fixed rigid endoport for initial hematoma evacuation (Figure 3D), then replaced with the FATSE (Figure 3E), which allowed dynamic sheath adjustment (8–16 mm diameter) to match hematoma geometry—starting collapsed (3–4 mm) for minimal disruption and gradually expanding during evacuation.

### Additional surgical techniques

Sheath Fabrication and Insertion: The FATSE sheath is fabricated from sterile, medical-grade polyvinyl chloride (PVC) sourced from saline bags. Strips are cut into 6 cm x 6 cm segments, rolled tightly into a compact sleeve (3–4 mm diameter) using tweezers, and inserted into the hematoma cavity via a rigid endoport. Upon removal of the endoport, the sheath expands naturally to form a tubular channel (8–16 mm diameter), adjustable intraoperatively by gentle manipulation to match the hematoma’s geometry.

### Tips for hematoma evacuation

For multi-compartmental hematomas, we recommend a ‘sheath-in-sheath’ technique, where a smaller secondary sheath (4–6 mm) is inserted through the primary sheath to access difficult-to-reach areas, such as intraventricular extensions or deep parenchymal clots. For cases with intraventricular involvement, the sheath can be angled toward the ventricles while maintaining a stable corridor to minimize parenchymal injury. Surgeons should adjust the sheath diameter gradually during evacuation to optimize visualization and avoid excessive retraction.

### Hemostasis and closure

Hemostasis is achieved under direct endoscopic visualization using low-power bipolar coagulation (10-15 W) (Figures 3F,G), with favorable post operative intracranial pressure reduction (Figure 3H). Care should be taken to avoid over-coagulation, which may damage surrounding tissue. Post-evacuation, a soft drainage catheter is placed to prevent reaccumulation, followed by layered closure with bone flap replacement (Figure 3I) to minimize cosmetic defects and support recovery. The mean procedure duration was 1 h 9 min 42 s (Figure 3J).

This protocol combined rapid ICP control with complete clot removal while minimizing parenchymal injury, leveraging the FATSE system’s adaptability for irregular hematoma morphologies.

## Results

The 54 patients in this study included 32 men and 22 women (1.45:1 ratio) with a median age of 56 years (range 42–71 years). Among these patients, there were 24 cases in Group A (basal ganglia with multi-regional extension, 44.4%), 18 cases in Group B (lobar with intraventricular extension, 33.3%), nine cases in Group C (thalamic with brainstem/ventricular extension, 16.7%), and three cases in Group D (isolated intraventricular hemorrhage with cast formation, 5.6%) (Table 1).

**Figure 4:**
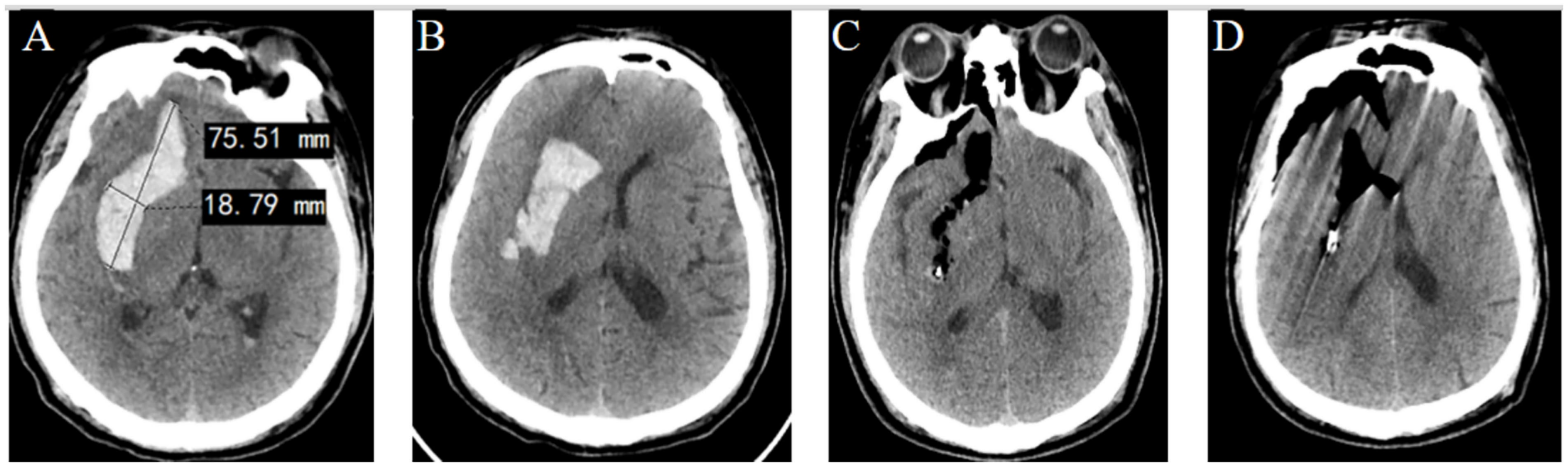
Pre- and post-operation CT scans of an irregular basal ganglia hemorrhage that was evacuated by FATSE combined with a “Two-in-One” neuroendoscopic approach. **(A)** Pre-operation CT scan showing the irregular “S” shaped hematoma located in the right basal ganglia with an anterior–posterior diameter of approximately 9.7 cm and lateral to medial diameter of 3.5 cm with a width-to-length ratio <50%. **(B)** The hematoma shaped was irregular. **(C)** Postoperative CT scan show route of evacuated hematoma was the same of preoperative hematoma shape. **(D)** Postoperative CT scan showed that the hematoma was almost completely evacuated.

**Figure 5:**
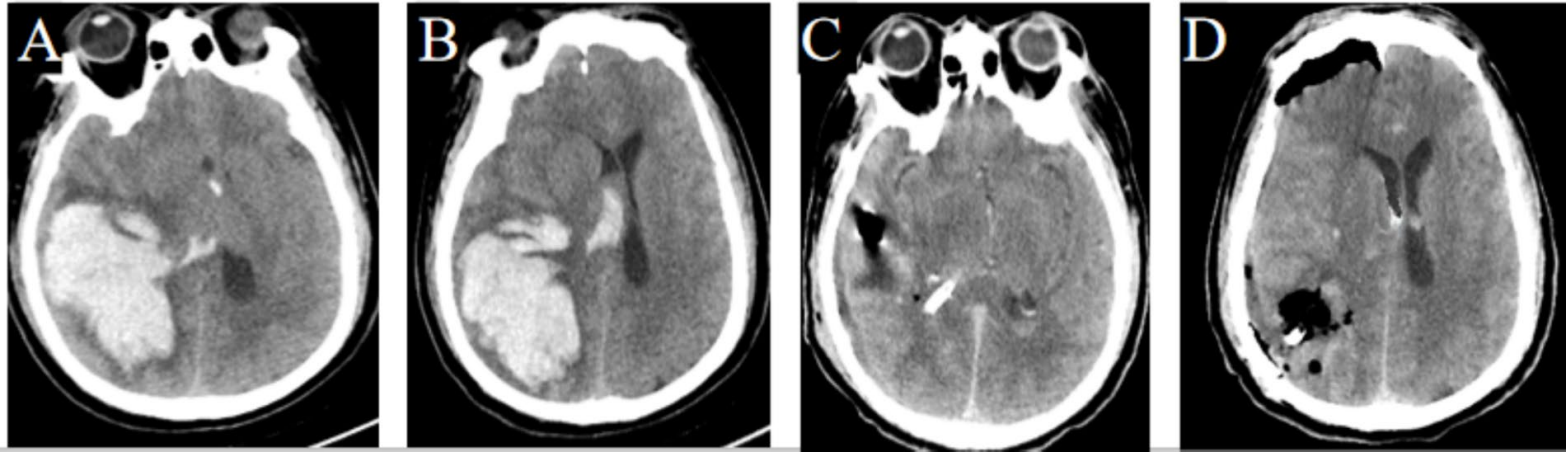
Pre- and post-operation CT scans of a huge irregular subcortical hemorrhage that was evacuated by FATSE combined with a “Two-in-One” neuroendoscopic approach. **(A)** Preoperative imaging demonstrates a huge irregular right temperoparietal hematoma. **(B)** CT scan show hematoma with intraventricular extension, causing significant midline shift. **(C)** Postoperative CT confirms near-complete hematoma evacuation in the temperoparietal lobe. **(D)** Postoperative CT scan shows hematoma in the lateral ventricle was also removed completely.

**Figure 6:**
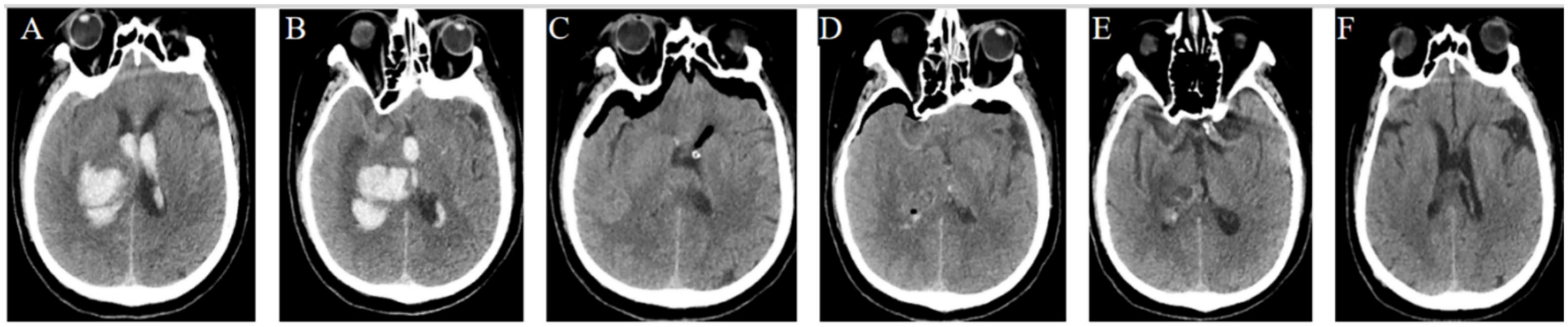
Pre- and post-operation CT scans of an irregular thalamic hemorrhage breaking into the ventricles that was evacuated by FATSE combined with a “Two-in-One” neuroendoscopic approach. **(A)** A CT scan collected upon admission showing a hematoma in the right thalamus and the expansion of the hematoma that broke into the lateral ventricles. **(B)** The CT scan showing hematoma extension into the third ventricles. **(C)** Postoperative CT scan showing that the hematoma in the thalamus and lateral ventricles almost completely evacuated. **(D)** CT scan showing hematoma in the third ventricles were removed successfully. **(E)** Postoperative 2 weeks CT image showing good results. **(F)** CT image showing no hydrocephalus after 2 weeks.

**Figure 7:**
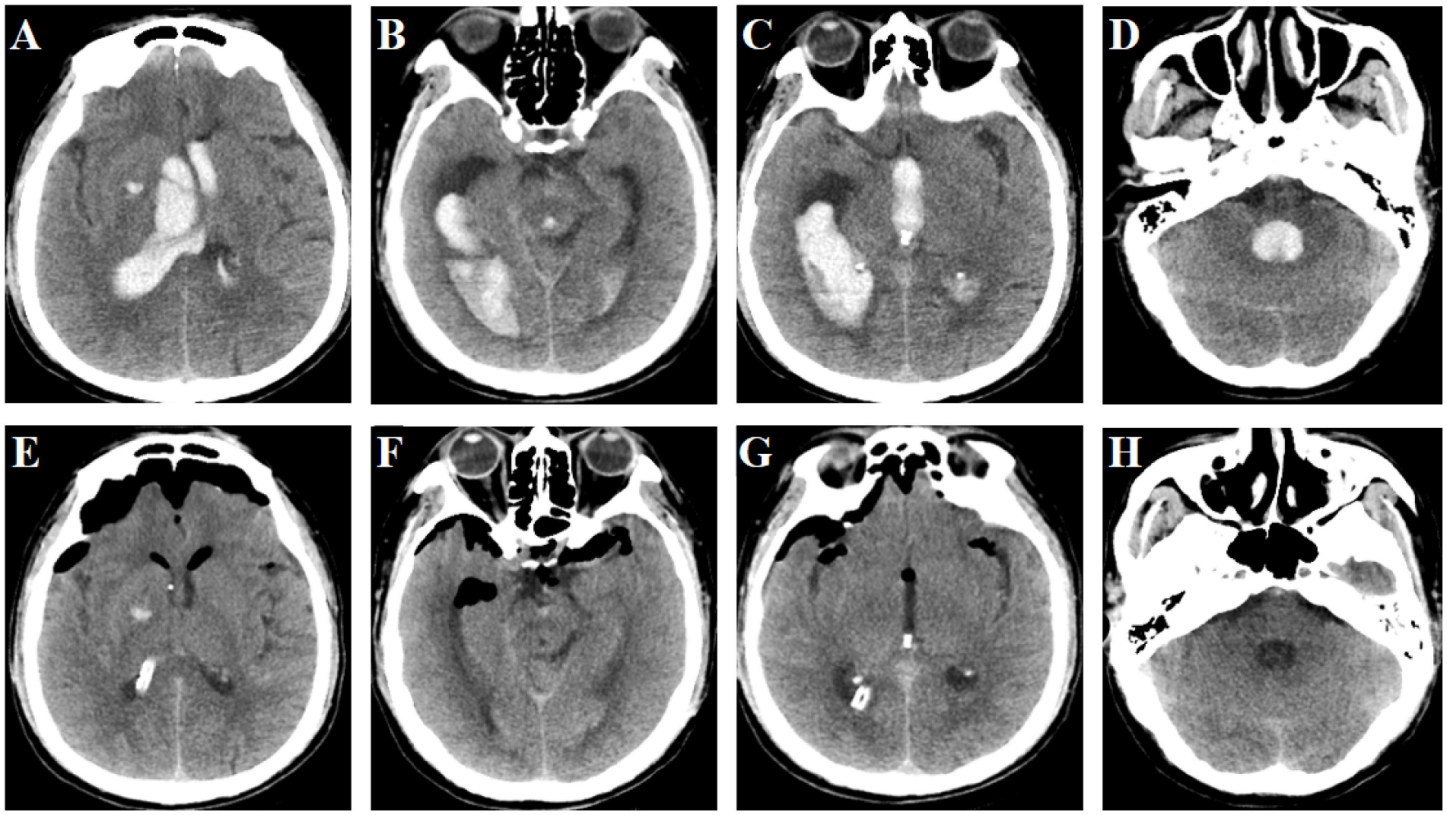
Pre- and post-operation CT scans of an irregular intraventricular hemorrhage that was evacuated by FATSE combined with a “Two-in-One” neuroendoscopic approach. **(A)** Preoperative CT scan showing the hematoma was located in the body of the lateral ventricles. **(B)** Preoperative CT scan showing the hematoma was extended to temporal horn of lateral ventricles. **(C)** The hematoma extended to the third ventricle. **(D)** The hematoma extended to the fourth ventricle. **(E)** CT scan showing the hematoma was completely evacuated in the body of the lateral ventricles. **(F)** CT scan showing evacuation of hematoma in temporal horn of lateral ventricles. **(G)** CT scan showing the hematoma was completely evacuated in the third ventricle **(H)** CT scan showing the hematoma was completely evacuated in the fourth ventricle.

In Group A, a temporal or frontal approach was selected based on hematoma extension, with post-operative CT scans indicating near-complete hematoma evacuation by the FATSE combined with the “two-in-one” neuroendoscopic approach. Follow-up CT scans confirmed favorable recovery based on imaging (Figures 4A–D). In Group B, the shortest trajectory corridor to the hematoma was utilized, achieving satisfactory evacuation while preserving eloquent cortex (Figures 5A–D). In Group C, a transventricular approach via the ipsilateral Kocher’s point served as the entry point, with follow-up CT scans showing no additional parenchymal damage to the thalamus (Figures 6A–F). In Group D, the ipsilateral Kocher’s point was also selected, and post-operative CT scans demonstrated complete clearance of ventricular casts (Figures 7A–H).

The preoperative hematoma volume was 58.4 mL (range 26.8–112.5 mL), and the average hematoma evacuation rate was 95.0 ± 0.8%. The highest hematoma evacuation rate was achieved in Group B (95.2 ± 0.7%), the second highest in Group A (95.1 ± 0.9%), the third highest in Group D (94.9 ± 0.5%), and the lowest in Group C (94.8 ± 1.1%).

All procedures were successfully completed, and the average surgery time ranged from 47 to 95 min with a mean length of 68 ± 12 min. The median Glasgow Coma Scale (GCS) scores were 7.2 ± 1.4 before surgery and 12.4 ± 1.8 at discharge; thus, the average GCS score improvement was 5.2 points.

No patients died during hospitalization, resulting in a surgical mortality rate of 0%. However, three patients (5.6%) required tracheostomy for prolonged ventilation but were successfully weaned. One patient (1.9%) in Group A experienced rebleeding due to uncontrolled hypertension 48 h post-operatively (hematoma volume 18 mL), which was managed successfully with endoscopic re-exploration. The rebleeding rate was 1.9%. Three cases (5.6%) developed pneumonia, treated effectively with targeted antibiotics, and two cases (3.7%) had transient electrolyte imbalances corrected medically. There were no cases of intracranial infection or device-related complications.

## Discussion

The management of irregular intracerebral hemorrhage has long posed significant challenges in neurosurgical practice (14). Our study introduces the FATSE system with a “two-in-one” neuroendoscopic approach as a comprehensive solution, demonstrating superior outcomes across all measured parameters. The key innovation lies in addressing three fundamental limitations of conventional techniques: restricted access to complex hematoma geometries (15), compromised visualization in deep or multi-compartmental clots (16), and prohibitive costs of specialized equipment (17). By combining real-time adjustable sheath diameter (8-16 mm) with integrated stereotactic decompression and endoscopic evacuation, we achieved a remarkable 95.0% mean evacuation rate across all irregular ICH subtypes, including challenging thalamic (94.8%) and intraventricular (94.9%) hemorrhages that traditionally show poorer outcomes with rigid endoport systems (10).

When contextualized against existing minimally invasive approaches, the FATSE demonstrates clear advantages. Compared to the SCUBA technique’s 88.7–93% evacuation rates (11), our method provides more complete clot removal while eliminating the need for specialized irrigation systems. Similarly, while Apollo/Artemis systems achieve 85–90% evacuation for supratentorial hemorrhages, they remain cost-prohibitive and less effective for intraventricular extension without adjunctive thrombolytics (17). The FATSE’s negligible material cost and comparable efficacy represent both a technical and economic advancement. This is particularly significant given that our cohort included patients with larger hematoma volumes (median 58.4 mL, range 26.8–112.5 mL) and more severe presentations (mean admission GCS 7.2) than many previous studies (18), yet still achieved 0% mortality and minimal complications (1.9% rebleeding, 5.6% pneumonia).

### Comparison with standard non-surgical management

Standard non-surgical management of ICH typically involves medical stabilization, aggressive blood pressure control, and monitoring for complications such as cerebral edema or hydrocephalus. However, for patients with large or irregular hematomas, non-surgical approaches are associated with mortality rates ranging from 30 to 50% and poorer functional outcomes compared to surgical interventions in select cases (2, 19). In our study, the FATSE approach achieved a 0% mortality rate and a mean GCS improvement of 5.2 points, suggesting potential advantages over non-surgical management, particularly for complex hematomas. These outcomes underscore the need to consider minimally invasive surgical options in cases where conservative treatment may be insufficient.

### Limitations and future directions

While the zero mortality rate in our cohort of 54 patients is encouraging, it should be interpreted within the context of a retrospective, single-center study focused on the technical aspects and preliminary outcomes of the FATSE approach. This finding is promising but preliminary, and we acknowledge the absence of statistical analysis as a limitation due to the descriptive nature of this initial report. A case–control or prospective study is necessary to rigorously evaluate the effectiveness of this method compared to existing treatments and to provide statistical power to confirm these outcomes. Additionally, the small sample size for Group D (n = 3) and the lack of long-term functional outcome data are limitations that warrant further investigation.

The clinical implications of these findings are substantial. First, the “two-in-one” approach’s rapid ICP reduction phase (3–5 min) addresses the critical time sensitivity in ICH management (20), while the subsequent endoscopic phase allows meticulous hemostasis—a combination that explains our lower rebleeding rate compared to conventional neuroendoscopy (1.9% vs. 2.4%) (8). Second, the sheath’s dynamic adjustability enables tailored treatment for each hematoma’s unique morphology, as evidenced by Group B’s 95.2% evacuation rate in lobar hemorrhages versus Group C’s 94.8% in thalamic cases. This adaptability is further demonstrated in Patient #9’s remarkable outcome (98.3% evacuation of a 41.6 mL basal ganglia-ventricular hemorrhage), showcasing the system’s potential when optimally applied.

Several important limitations warrant consideration. The retrospective design and single-center experience may affect generalizability. The small Group D sample (n = 3) particularly limits definitive conclusions about pure intraventricular hemorrhages, though our 94.9% evacuation rate in these cases remains promising. Additionally, the 1-month follow-up precludes assessment of long-term functional outcomes, an important metric for surgical ICH interventions.

Future research directions should prioritize three key areas: (1) multicenter randomized trials comparing FATSE directly against SCUBA and Apollo/Artemis systems with 6-month functional outcomes, (2) health economic analyses to quantify the system’s cost-saving potential across different healthcare settings, and (3) technological refinements such as augmented reality integration for trajectory planning and pressure-sensitive sheath modifications. The system’s adaptability also suggests potential applications beyond ICH, including tumor biopsies and cyst fenestrations, which merit exploration.

## Conclusion

The FATSE system demonstrates promise in achieving high evacuation rates (95.0%) and favorable neurological recovery (ΔGCS + 5.2) for irregular ICH, with a 0% mortality rate in this cohort. However, these results require validation through controlled studies to establish comparative efficacy and generalizability.

## Data Availability

The original contributions presented in the study are included in the article/Supplementary material, further inquiries can be directed to the corresponding authors.

## Footnotes

^1^ http://www.slicer.org
